# The hidden burden of adolescent pregnancies in rural Sri Lanka; findings of the Rajarata Pregnancy Cohort

**DOI:** 10.1186/s12884-021-03977-1

**Published:** 2021-07-07

**Authors:** Thilini Chanchala Agampodi, Nuwan Darshana Wickramasinghe, Hemali Gayathri Jayakodi, Gayani Shashikala Amarasinghe, Janith Niwanthaka Warnasekara, Ayesh Umeshana Hettiarachchi, Imasha Upulini Jayasinghe, Iresha Sandamali Koralegedara, Sajaan Praveena Gunarathne, Dulani Kanchana Somasiri, Suneth Buddhika Agampodi

**Affiliations:** 1 Department of Community Medicine, Faculty of Medicine and Allied Sciences, Rajarata University of Sri Lanka, Saliyapura, 50008 Sri Lanka; 2 Regional Director of Health Services Office, Anuradhapura, Sri Lanka; 3 Department of Anatomy, Faculty of Medicine and Allied Sciences, Rajarata University of Sri Lanka, , Saliyapura, Sri Lanka

**Keywords:** Adolescent pregnancies, Pre-conceptional care, Sexual and reproductive health services, Sri Lanka

## Abstract

**Background:**

Adolescent fertility is a main indicator of the Sustainable Developmental Goal (SGD) three. Although Sri Lanka is exemplary in maternal health, the utilization of Sexual and Reproductive Health services (SRH) by adolescents is less documented. We describe the hidden burden, associated biological and psychosocial factors and utilization patterns of pre-conceptional services among pregnant adolescents in rural Sri Lanka.

**Methods:**

The study is based on the baseline assessment of the Rajarata Pregnancy Cohort (RaPCo) in Anuradhapura. Pregnant women newly registered from July to September 2019 were recruited to the study. The period of gestation was confirmed during the second follow-up visit (around 25–28 weeks of gestation) using ultra sound scan data. A history, clinical examination, anthropometric measurements, blood investigations were conducted. Mental health status was assessed using the Edinburgh Postpartum Depression Scale (EPDS).

**Results:**

Baseline data on gestation was completed by 3,367 pregnant women. Of them, 254 (7.5%) were adolescent pregnancies. Among the primigravida mothers (*n* = 1037), 22.4% (*n* = 233) were adolescent pregnancies. Maternal and paternal low education level, being unmarried, and less time since marriage were statistically significant factors associated with adolescent pregnancies (*p* < 0.05). Contraceptive usage before pregnancy, utilization of pre-conceptional health care services, planning pregnancy and consuming folic acid was significantly low among adolescents (*p* < 0.001). They also had low body mass index (*p* < 0.001) and low hemoglobin levels (*p* = 0.03). Adolescent mothers were less happy of being pregnant (*p *= 0.006) and had significantly higher levels of anxiety (*p* = 0.009).

**Conclusion:**

One fifth of women in their first pregnancy in this study population are adolescents. Nulli-parous adolescents exert poor social stability and compromised physical and mental health effects. The underutilization and/or unavailability of SRH services is clearly associated with adolescent pregnancies.

## Introduction

Adolescent fertility is considered as a challenging public health problem in the context of the growing adolescent population in South East Asian and African regions [1]. There are substantial intercountry, as well as intra-regional disparities in adolescent fertility rates [2]. While universal health care is a key to reduce inequalities in health [3], even countries with successful public health systems have failed in reducing the adolescent pregnancy rates [4, 5].

Social inequalities, as a result of diverse individual, socio-cultural, environmental, economic, and health system related factors, could lead to high rates of adolescent pregnancies [6, 7]. Adolescent pregnancies are associated with maternal and feto-infant complications, such as low fat or lean body mass, risk of iron and calcium deficiency leading to maternal anemia, hypertensive disorders, as well as poor fetal growth, and low birth weight of newborns [8, 9]. The effects on maternal and fetal nutrition depict childhood stunting as well as poor complementary feeding practices [10, 11] leading to a vicious cycle of intergenerational malnutrition. Prevention of adolescent pregnancies needs multisectoral resources and human development [12]. Hence, adolescent fertility rate is identified as a major indicator of Sustainable Developmental Goal 3.

In order to effectively address the public health issue of adolescent pregnancies, the health, education, and social care sectors need to act on two main aspects; prevention of unintended adolescent pregnancies and empowering adolescents that intend to be pregnant (considering the cultural influences) or optimizing the health status to prepare for the pregnancy and childbearing in those who are pregnant [4]. Since adolescent pregnancies are not amenable to simple interventions as they have either short term or modest effects [13], multisectoral complex approaches are suggested in global literature [14]. Although empowering girls have been identified as a key factor in the prevention of adolescent pregnancies [15], global as well as regional evidence on how the prevailing health systems are utilized by adolescents before and after conceiving is scarce.

Masked by the cultural acceptance, the “perceived threat” of adolescent pregnancies is low compared to other maternal health problems in the Sri Lankan context. Causes and implications of adolescent pregnancies in Sri Lanka are similar to those reported in the global literature [16, 17] except for satisfactory family support received during pregnancy [14]. However, the adolescent fertility rate has been stagnated around 30 per 1,000 adolescents during the past four decades in Sri Lanka [18], which raises concerns about the effectiveness of the existing interventions. National data on teenage pregnancies reveal a prevalence of 4.4%, which has shown very slow improvement over the past few years amounting to around 20% of teenage pregnancies occurring among adolescents below 17 years [19].

During the past two decades, several changes to the legislative framework were made concerning the adolescent health and education systems. Provision of Adolescent and Youth Friendly Health Services (AYFHS) were mediated by the national focal point of maternal and child health, the Family Health Bureau, Ministry of Health Sri Lanka. Initiated in 2005, the AYFHS has been strengthened during the past decade to establish a network of routine public health services including clinic and field care by primary health care officers [18]. Sexual and reproductive health (SRH) education was incorporated into the school curriculum commencing from early adolescence. The maternal care program was streamlined with an additional service package for newly married couples [20] (facilitated by a five-item tool kit: The invitation card; pre-conceptional screening tool; Body Mass Index (BMI) calculator and two health educational booklets). All couples at the registration of their marriage are informed by the marriage registrars to utilize the services. The primary health care officers at the field level register all newly married couples and direct them to a pre-conceptional clinic and two pre-conceptional sessions held (classes) focused on risk assessment, care provision, and health education [21] The Medical Officer of Health (MOH) in charge of the particular health administrative are is responsible for implementation and monitoring of the services. The medical, psychosocial, and environmental risk factors of health and wellbeing are identified using the screening tool which is filled by the female, the public health midwife (PHM), and the MOH. Clinical examination, assessment of Body Mass Index (BMI), and blood investigations are performed. Care provision includes family planning services, rubella vaccination, and folic acid supplementation. During the sessions, where both husband and wife have to participate, health education on nutrition, sexual and reproductive health, and psychosocial wellbeing promotion is delivered.

Although the policy guidelines and implementation are initiated at the central level educational and health authorities, the effectiveness of these services on promoting adolescent SRH is not well evaluated. There is a gap in the local evidence base in assessing the implementation gaps and the effectiveness of the impact of these changes. Further, the level of utilization of newly added educational and health service resources by adolescents are poorly documented. We describe here the hidden burden of adolescent pregnancies and utilization patterns of pre-conceptional services among pregnant adolescents in rural Sri Lanka.

## Methods

The Rajarata Pregnancy Cohort (RaPCo) was established in 2019 as the largest community-based pregnancy cohort in Sri Lanka. The details of the establishment and the methodology is published previously [22]. The methods in a nutshell are reported here. The study was conducted in Anuradhapura; the largest geographical district of the country. It is a predominantly rural area with a dry climate, agriculture-based economy and the resident population is 902,930 with a birth rate of 17.8/1000 population [19]. The total fertility rate in the Anuradhapura district is 2.4 and the median age at first birth is 23.9 years, which is one of the lowest in Sri Lanka [23]. In the Sri Lankan maternal health system, all pregnant women are routinely registered by the area PHMM, either in the field or during the booking visit (which is the first antenatal visit). Of the pregnant mothers registered in the area in 2015, 82.3% were registered in field clinics before the eighth week of pregnancy and 96.0% had at least one clinic visit before delivery [24].

From July to September 2019, all pregnant women newly registered in the district maternal solution was achieved; anhedonia, anxiety, and depressionhealth program were invited to participate in the RaPCo study. All newly registered pregnant women with a period of gestation less than 12 weeks [either by LRMP or by ultrasound scan (USS) if and when available during the baseline study and the follow up] were directed to a special clinic, in which they were recruited to the study. During the special RaPCo clinic, a detailed history, clinical examination, anthropometric measurements and routine blood investigations were carried out by a team of researchers, including a fully qualified, registered physician, MBBS qualified medical graduates, and trained research assistants (third-year medical undergraduates).

Referrals and participation in the study were cross-checked regularly with the public health system to ensure data quality. An interviewer-administered questionnaire was used to collect socio-demographic, medical, and obstetric data. A self-administered questionnaire was used to collect details on mental health and wellbeing while awaiting the clinical examination. This included a mental wellbeing component and the validated Sinhala and Tamil versions of the Edinburgh Postpartum Depression Scale (EPDS). Ethical clearance was obtained from the ethics review committee of the Faculty of Medical and Allied Sciences, Rajarata University of Sri Lanka.

## Measures and definitions

For the present study, the adolescent period was defined as age 10 to 19 years. Age at conception was calculated using the date of birth. In a few instances where the date of birth was missing, the mother reported age in completed years was documented. The period of gestation at the registration among women with uncertain dates and irregular periods were retrospectively calculated using USS data available in the pregnancy record during the cohort follow-up visits at 24–28 weeks. The BMI and the anemia status were assessed to describe the maternal physical health status. BMI was interpreted according to the Asia Pacific cut-off values [25]. Anemia classification was based on the recommended World Health Organization (WHO) cut-off values for the first trimester [26]. The mental health status was assessed by the Sinhala and Tamil versions of EPDS scores, validated for the use during antenatal period [27] and the perceived feeling of happiness of being pregnant. The factor structure of the EPDS RaPCo study was previously analyzed and a three-factor solution was achieved; anhedonia, anxiety, and depression [28]. We used these factor scores for this study. The preparation for pregnancy was assessed by including questions on awareness and utilization of pre-pregnancy health services offered by the routine maternal health service delivery in the district.

## Results

Of the 3,374 pregnant women participated in clinics, baseline data on gestation was fully completed for 3,370 pregnant women (response rate 99.9%). The mean age and standard deviation (SD) of the study participants was 27.9 (5.6) years. Of them, 254 (7.5%) were adolescent pregnancies. Even though only women with POA less than 12 weeks were invited, 168 with POA more than 12 weeks and 62 with uncertain dates at the time of recruitment also visited the recruitment clinic. We observed that only 89.1% of the adolescents compared to 95.4% of non-adolescent pregnant women registered with a POA less than 12 weeks (Chi-square 19.0, *p* < 0.001).

### Characteristics of the adolescent pregnant women

Among adolescent pregnant women, 4 (0.1%), 19 (0.6%), 41 (1.2%), 89 (2.6%) and 101 (3.0%) were aged 15, 16, 17, 18 and 19 years respectively. The distribution of adolescent pregnancies by gravidity shows that 233 (91.9%) were primigravidae. Of the others, 20 (1.7%) were in their second pregnancy (Table 1).

**Table 1:** Distribution of adolescent and other pregnancies by parity in the RaPCo

	**Adolescent pregnancies**		**Other pregnancies**		**Total**
	**n**	**%**	**n**	**%**	
First pregnancy	233	22.4	808	77.6	1,041
Second pregnancy	20	1.9	1,039	98.1	1,059
Third pregnancy or above	1	0.1	1,269	99.9	1,270
Total	254	7.5	3,116	92.5	3,370

Since 91.7% (*n* = 233) of adolescent pregnancies were primigravidae, we selected the cohort of pregnant women in their first pregnancy (*n* = 1,041) for further comparisons to assess the burden and the type of care they received during the pre-pregnancy period.

### Characteristics of the primigravida women

We compared the demographics of primigravida adolescents with non-adolescent primigravida women. The mean (SD) age of the primigravida mothers was 23.6 (4.6) years. This sample had only 16 (1.5%), pregnant mothers, with the highest education level of grade 8 or below. Another 494 (47.7%) dropped out of school in between grades 9 and 11. A degree was completed by 159 (15.2%) participants. The ethnic composition of the sample was; 916 (88.0%) Sinhalese, 113 (10.9%) Moor/Malay, and 12 (1.2%) other ethnic groups. Of the 1041 selected, 60 (5.8%) were not legally eligible to marry (< 18 years) at the time they have participated in the study. Of the married women (n = 983), 331 (33.7%) were within the first six months, 252 were within 6–12 months and 400 (40.7%) were after 12 months of marriage.

### Primigravida adolescent pregnancies

Among primigravida pregnant women, the distribution of adolescent pregnancies was not significantly different across ethnic groups (Table 2). However, maternal and paternal education level, marital status, and time since marriage were statistically significant factors associated with adolescent pregnancies (*p* < 0.05). As expected, maternal education was low among adolescent pregnant mothers compared to others. Similarly, yet more significant pattern was observed concerning paternal education. Of the 58 unmarried mothers in this sample, 49 (85.9%) were adolescent mothers. A higher proportion (77%) of adolescent mothers conceived for the first time within 12 months after marriage.

**Table 2:** Characteristics of primigravida women

	**Adolescent pregnancies**		**Other pregnancies**		**Total**	**Significance**	
	**n**	**%**	**n**	**%**			
**Ethnicity (*N* = 1041)**							
Sinhala	206	88.4	710	88.1	222	Chi-Square	0.085
Moor/Malay	24	10.3	89	10.8	32	*p*	0.958
Other	3	1.3	9	1.1	3		
**Highest educational level—pregnant woman (*N* = 1036)**							
Up to Grade 10	27	11.3	40	4.9	36	Chi-Square	152.909
Grade 11	169	73.5	270	33.8	178	*p*	** < 0.001**
Grade 12–13	35	15.2	490	61.4	38		
**Highest educational level—husband (*N* = 1033)**							
Up to Grade 10	32	13.4	47	5.6	39	Chi-Square	105.909
Grade 11	176	76.2	376	47.0	187	*p*	** < 0.001**
Grade 12–13	24	10.4	378	47.4	27		
**Self-reported formal sexual education (*N* = 1036)**							
Received	122	52.4	505	62.9	625	Chi-Square	8.340
Not received	110	47.6	299	37.1	407	*p*	**0.003**
**Marital status (*N* = 1041)**							
Currently married	182	78.4	801	99.1	980	Chi-Square	148.304
Currently not married	51	21.6	7	0.9	57	*p*	** < 0.001**
**Duration since marriage (*N* = 983)**							
6 months or less	80	44.0	248	31.1	328	Chi-Square	30.951
7–12 months	61	33.5	191	23.9	252	*p*	** < 0.001**
More than12 months	41	22.5	359	45.0	400		

**Fig. 1:**
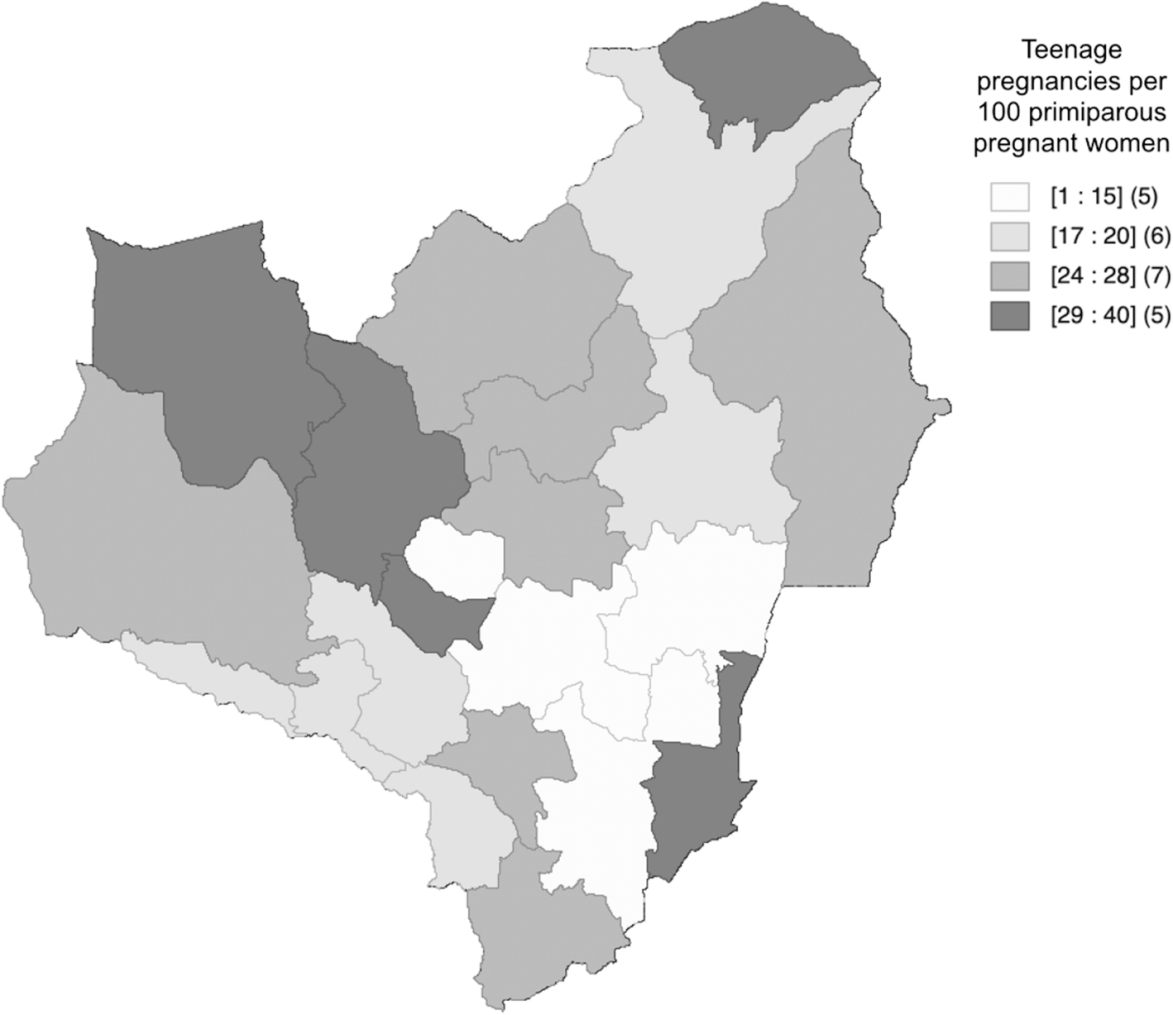
Distribution of adolescent pregnancies among nulli-parous women in Anuradhapura district

Geographical distribution of primigravida adolescent pregnancies as a percentage of primigravida pregnancies shows a wide variation ranging from 12.2% (*n* = 9/75) in Kekirawa to 40.0% (*n* = 6/15) in Palugaswewa (Fig. 1). It is noteworthy to observe that in 50% of MOH areas, one in four or more primigravida pregnancies are adolescent pregnancies. The MOH areas were Padaviya (*n* = 8, 33.3%), Nuwaragampalatha Central (*n* = 25, 32.5%), Nachchaduwa (*n* = 8, 30.8%), Mahawilachchiya (*n* = 7, 29.2%), Horowpathana (*n* = 11, 28.2%), Nochchiyagama (*n* = 18, 28.1%) Ipalogama (*n* = 14, 27.5%), Rambawa (*n* = 11, 26.8%), Palagala (*n* = 11, 25.6%) and Madawachchiya (*n* = 17, 25.0%).

### Planning and preparation for pregnancy among primigravida women

We looked at routinely available health services for all eligible couples through the maternal health care system in Sri Lanka to assess the preparation for pregnancy (Table 3). Out of the services analyzed, it is important to note that even though the services are freely available, contraceptive use, pre- and post-conceptional folic acid use, attendance to pre-conceptional educational sessions were poor among adolescent females in comparison to their counterparts (*p* < 0.05).

**Table 3:** Planning and preparation for pregnancy among of adolescent and other pregnancies among primigravida women

	Adolescent pregnancies		Non-adolescent pregnancies		Significance	
	n	%	n	%		
Contraceptive use (*N* = 1037)						
Yes	109	46.8	251	31.2	Chi-Square	19.301
No	124	53.2	553	68.8	*p*	< 0.001
Planned pregnancy (*N* = 1030)						
Yes	121	52.4	646	84.2	Chi-Square	76.388
No	110	47.6	153	19.1	*p*	< 0.001
Attending pre-pregnancy sessions						
Yes, with husband	33	14.2	162	20.0	Chi-Square	74.137
Yes, alone	6	2.6	67	8.3	*p*	< 0.001
No	27	11.6	171	21.2		
Not aware	47	20.2	222	27.5		
No answer	110	49.3	153	23.0		
Screened for anemia in pre-pregnancy sessions (N 179)^**a**^						
Yes	14	46.7	62	41.6	Chi-Square	4.190
No	16	53.3	87	58.4	*p*	0.123
Cardiovascular examination in pre-pregnancy sessions (176)^**a**^						
Yes	6	20.0	37	25.3	Chi-Square	4.017
No	24	80.0	110	74.7	*p*	0.134
Pre-conceptional folic acid use						
Yes	79	33.9	532	65.8	Chi-Square	85.616
No	41	17.6	109	13.5	*p*	< 0.001
Not answered	113	48.5	167	20.7		
Folic acid use after conception						
Yes, frequently	62	26.6	453	56.1	Chi-Square	79.281
Yes, infrequently	10	4.3	56	6.9	*p*	< 0.001
No	5	2.1	20	2.5		
Not answered	156	67.0	279	34.5		

N was given when there are missing values or when the missing data are not included in the table

^a^Only among those who participated in pre-pregnancy sessions

Of the 23 mid-adolescents (age 15–16 years), 18 (78.3%) reported that the pregnancy was unplanned, whereas 92 (44.2%) out of 208 late-adolescents (17–19 years) reported the pregnancy was planned. The number of adolescents got pregnant while they were in school or within six months after the completion of the last grade was 55 (23.3% of all adolescents) while 181 (76.7%) became pregnant more than six months after leaving school.

### Health status of primigravida women

The health statuses of adolescent mothers in comparison to their counterparts were assessed (Table 4). There were significant differences in the groups concerning their BMI, first-trimester hemoglobin, and also general feeling towards the current pregnancy (*p* < 0.05).

**Table 4:** Comparison of the health status of adolescent and other pregnancies among primigravida women

	Adolescent pregnancies		Non-adolescent pregnancies		Significance	
	n	%	n	%		
BMI (kg/m^2^)						
< 18.5	81	35.5	156	20.0	Chi-Square	29.656
18.5–22.9	87	38.2	296	37.8	*p*	< 0.001
> = 23	60	26.3	331	42.2		
Total	228		783			
Hemoglobin (g/dl)						
> = 11	190	81.8	704	87.4	Chi-Square	6.979
10–10.9	28	11.9	79	9.5	*p*	0.03
8–9.9^a^	14	5.9	25	3.2		
< 8^a^	1	0.5	0	0.0		
Total	233		808			
Feeling about pregnancy						
Very happy	179	84.8	668	92.2	Chi-Square	10.109
Somewhat happy^a^	20	9.5	47	6.2	*p*	0.001
Same as pre-pregnancy^a^	5	2.4	3	0.4		
Unhappy^a^	1	0.5	0	0.0		
Extremely unhappy^a^	6	2.8	9	1.2		
Total	211		747			

^a^These categories were amalgamated within the question for statistical testing

A fully completed EPDS questionnaire was available for 1,003 of 1,037 primigravida women. We compared the EPDS derived factors between adolescents and other pregnancies (Table 5). The EPDS in the original cohort showed three extracted factors; anhedonia, anxiety, and depression [28]. adolescent pregnant women had significantly higher mean scores (t = 3.202, *p* < 0.001) for anxiety factor.

**Table 5:** Comparison of anhedonia, anxiety, and depression factor scores

Factor	Group	N	Mean	Std. Deviation	Std. Error mean	t	df	*p*
EPDS Factor score 1 anhedonia	Adolescent	221	0.081	0.914	0.061	0.813	1001	0.416
	Others	782	0.024	0.928	0.033			
EPDS Factor score 2 anxiety	Adolescent	221	0.124	0.949	0.064	2.614	1001	0.009
	Others	782	-0.043	0.801	0.029			
EPDS Factor score 3 depression	Adolescent	221	-0.024	0.817	0.055	0.737	1001	0.461
	Others	782	-0.064	0.665	0.024			

## Discussion

Contrary to the perceived ‘satisfaction’ among healthcare professionals as having only 4.4% adolescent pregnancies [24] in Sri Lanka, this large community-based study representing a whole district reveals that adolescents accounted for one-fifth of primigravida pregnancies in Anuradhapura. Underutilization and or inadequacy of pre-conceptional care and SRH education and the need for keeping the adolescents in school are highlighted in this study.

During the past five years, the Sri Lankan national statistics suggest that the percentage of adolescent pregnancies has been gradually declining from 5.2% in 2015 to 4.4% in 2019 [21]. However, in the total sample of pregnant women enrolled in the RaPCo study, the corresponding percentage is 6.9%, which is higher than the recent national and subnational level estimates [19]. The percentage of primigravida mothers in the RaPCo study was 30.8%, which is approximately similar to the corresponding national value of 32.0%; thus, the high rate in this sample could not be attributed to differences in parity. Besides, we observed that there is a gross variation of percentages across health administrative areas (MOH areas) where more than 50% of the areas had one in four primigravida pregnancies being among adolescents. Hence, these findings urgently warrant action.

The socio-demographic characteristics of pregnant adolescents in this sample are similar to those that are documented in global, regional, and local studies [7, 11, 29]. In a similar vein, our study findings suggest a significant association of low educational level with adolescent pregnancies. It is expected to have a low level of education since they are still young; yet, our analysis shows that a significantly higher number has not completed Grade 11, which is usually completed at the age of 16 years. Despite having a free education system in Sri Lanka, we observe that early dropouts from school are associated with adolescent pregnancies in the district. Interventions targeting to improve school retention and targeted SRH campaigns for school dropouts in rural areas are some suggestions to improve the situation.

In accordance with the available literature [8, 9], our study suggests that adolescent primigravida mothers are not physically and mentally ready for pregnancy or childbearing. Adolescent mothers had suboptimal BMI, high prevalence of anemia, and poor mental health status, which will have short and long-term effects on both mother and child [30] as well as on the family and society. The findings further emphasize the importance of optimizing the health status of females of the reproductive age group from the pre-conceptional level. Thus, the mechanisms for early identification, referral, and follow-up for health and nutritional problems among female adolescents need to be further strengthened.

In the free education system in Sri Lanka, SRH education is incorporated into the curriculum to be taught before mid adolescents [18]. Even though almost all (98.6%) primigravida mothers have completed education beyond Grade 8, in which basic SRH education is supposed to be taught at schools, 47.1% of adolescent mothers compared to 37% of older age group stated that they have not received any SRH before pregnancy. This reflects that despite universal SRH education, adolescents who have become pregnant have less grasped the concepts during their schooling. Given that a multitude of factors could contribute to the high prevalence of adolescent pregnancies, it is an opportune time to evaluate the efficiency of SRH education in the Sri Lankan school curriculum on students of different socio-cultural backgrounds. According to our study, 34.4% of primigravida adolescents were within six months of marriage. Considering the relatively high level of education in the sample, it is obvious that they conceived either while schooling or just after cessation of education. This reflects the importance of keeping the girls in schools and a comprehensive education sector response to preventing adolescent pregnancies. Hence the SRH education and youth-friendly Health service strategies need to be re-evaluated. Although this fact has been identified as an existing problem for almost two decades [31, 32], it seemed to be a neglected or inefficient area in service provision.

Sri Lanka has a well-established maternal care service package delivering accessible field clinic-based and domiciliary care by the area public health team targeted at pre-conceptional, antenatal as well as postnatal care [33]. These services include the provision of appropriate family planning services, risk assessment, and health promotion for all newly married couples. We observed that the question on pre-conceptional clinics is having very high level of missing data; the highest in all variables we included in this analysis. This may be suggestive of the fact that are not aware of such a clinic or could not understand the question due to lack of knowledge on such a clinic. With the available limited data, it shows that the service utilization by adolescents during the pre-conceptional period is significantly lower than the older pregnant women in this sample. It is noteworthy to observe that approximately half of the adolescent primigravida mothers (53.4%) have reported that they have not used any contraceptive method and also that the current pregnancy was unplanned. These results indicate a very high unmet need for family planning. The finding that a high percentage of adolescents conceive within twelve months implies that family planning services including counseling are not met to postpone pregnancy among them. However, according to the findings, the contraceptive usage is lower and the unplanned pregnancies are higher among non-adolescent pregnant women indicating a universal deficit in provision of family planning services. It is also noteworthy that around 17% of adolescent primigravida women have attended pre-conceptional clinics. The reasons for not facilitating postponement of pregnancy by health care providers at this stage need to be investigated further. The percentage of unplanned pregnancies are lower than previously reported in the Kandy district [34]. The different contexts, ethnic compositions and recent improvement of pre-conceptional care services could be the reasons.

Sri Lanka maternal care program had the “safe motherhood” clinics aiming at newly wedded married couples since the introduction of the safe motherhood program. Streamlining these services, the Sri Lankan maternal care program introduced focused pre-pregnancy sessions on important health issues for married couples [33]. Living together or married adolescents should be a prime target of pre-conception service package because of their high physical and mental vulnerability levels. However, both the low participation and lack of awareness of pre-pregnancy sessions raise concerns concerning the utilization of these sessions by adolescents in the target group. Availability and access to services and creating awareness of such services among adolescents could contribute to increasing participation. In addition, present strategies of inviting newly married couples to the pre-conceptional sessions need to be revised and expand to include couples who are living together and who intend to marry. Appropriate modes of communication should be used to attract vulnerable groups for the program to improve utilization.

In the view of reducing adolescent pregnancies, Sri Lanka needs to adopt innovative as well as effective multisectoral strategies for adolescent and youth development [14]. The findings of this study question the acceptability, awareness, and feasibility of preconception care services in the study area. Exploration of factors that lead to under-utilization of services needs to be addressed. It is of utmost importance to further assess the underlying root causes for not using freely available family planning methods, considering the aspects of awareness, availability, accessibility as well as acceptability. Focusing on micro geographical and ethnic disparities would be essential in planning interventions.

This study has several limitations. All pregnant women registered in the maternal care program of the Anuradhapura district were invited to participate in the study. Although the participation rate was very high as the special clinics that recruited participants were incorporated to the essential service provision pathway, we may have missed recruiting few mothers of the district. Although there is vey high coverage of the maternal care program of the Anuradhapura district [23], a very small percentage of women may have not been captured by the program. As the pre-conceptional services are not streamlined in the system as antenatal care services, service provision may not be the same universally within the district. For this reason, the pattern of pre-conceptional care services utilization should be cautiously interpreted and further investigated to distinguish between service availability and utilization.

## Conclusion

This study indicates that primigravida adolescent pregnancies are a significant public health problem in Anuradhapura district, Sri Lanka. Availability and awareness of AYFHS and pre-pregnancy care in the district need to be evaluated and strengthened given the low awareness and service utilization reflected in our study findings. There are lessons to be learnt with regard to the importance of meticulous exploration of even routinely available data to identify the existing service delivery and utilization gaps from a country with a well-established maternal and child health program.

## Data Availability

The datasets generated during the current study are be available from the corresponding author upon request.

## Abbreviations

SDG: Sustainable Developmental Goals
SRH: Sexual and reproductive health
RaPCo: Rajarata Pregnancy Cohort
EPDS: Edinburgh Postpartum Depression Scale
AYFHS: Adolescent and Youth Friendly Health Services
BMI: Body Mass Index
MOH: Medical Officer of Health
PHM: Public health midwife
POA: Period of gestation
USS: Ultra Sound Scan
WHO: World Health Organization
SD: Standard deviation
